# Effect of Intermittent Energy Restriction on Flow Mediated Dilatation, a Measure of Endothelial Function: A Short Report

**DOI:** 10.3390/ijerph15061166

**Published:** 2018-06-04

**Authors:** Michelle L. Headland, Peter M. Clifton, Jennifer B. Keogh

**Affiliations:** 1 School of Pharmacy and Medical Sciences, University of South Australia, Frome Road, Adelaide 5000, South Australia, Australia; michelle.headland@mymail.unisa.edu.au (M.L.H.); peter.clifton@unisa.edu.au (P.M.C.); 2 Alliance for Research in Exercise, Nutrition and Activity (ARENA), University of South Australia, Frome Road, Adelaide 5000, South Australia, Australia; 3 Sansom Institute for Health Research University of South Australia, Frome Road, Adelaide 5000, South Australia, Australia

**Keywords:** intermittent energy restriction, endothelial function, flow mediated dilatation

## Abstract

Intermittent energy restriction is a popular alternative to daily energy restriction for weight loss; however, it is unknown if endothelial function, a risk factor for cardiovascular disease, is altered by periods of severe energy restriction. The objective of the study was to determine the impact of two consecutive very low energy intake days, which is the core component of the 5:2 intermittent energy restriction diet strategy, on endothelial function compared to consecutive ad libitum eating days. The secondary objective was to explore the effects of these dietary conditions on fasting glucose concentrations. This was a 4-week randomized, single-blinded, crossover study of 35 participants. Participants consumed a very low energy diet (500 calories for women, 600 calories for men) on two consecutive days per week and 5 days of habitual eating. In weeks 3 and 4 of the trial, participants had measurements of flow mediated dilatation (FMD) and blood samples taken following either 2 habitual eating days or 2 energy restricted days in a randomized order. FMD values were not different after the two eating states (8.6% vs. 8.3%, *p* = 0.7). All other outcome variables were unchanged. Endothelial function, as measured by flow mediated dilatation, was not altered by two consecutive very low energy intake days. Further investigations assessing the impact in specific population groups as well as different testing conditions would be beneficial.

## 1. Introduction

Individuals who are overweight or obese are at an increased risk of developing cardiovascular disease [1] and other metabolic disorders including dyslipidemia, insulin resistance, and hypertension [2]. Obesity has also been linked to impaired endothelial function, which predicts atherosclerotic disease progression and rates of cardiovascular events [3,4]. A recent meta-analysis by Joris, P.J. et al. [5] concluded that weight loss can significantly improve fasting flow mediated dilatation (FMD) in adults; however, the effect may be influenced by subject characteristics, dietary composition and type of weight loss intervention. Intermittent energy restriction, a weight loss intervention gaining in popularity, has been shown to be as effective as the traditional moderate daily calorie restriction on weight loss, insulin sensitivity and cardio-metabolic biomarkers [6,7,8,9,10,11]. One such intermittent energy restriction strategy is the 5:2 diet, during which individuals consume a very low energy intake for two days per week (500 calories for women and 600 for men) combined with 5 days of habitual eating. Endothelial dysfunction is an indicator of future cardiovascular disease risk [12], which occurs very early in the atherosclerotic process and can be measured prior to clinical presentation of the disease [13]. Flow mediated dilatation (FMD) is a non-invasive and reliable technique used to assess endothelial dysfunction and correlates well with more invasive testing techniques, as well as the severity of atherosclerosis [14], and is a strong predictor of cardiovascular events in patients with established disease [15]. However, whether endothelial function as assessed by FMD is altered immediately following several days of intermittent energy restriction, as is performed during the 5:2 diet, remains unknown.

This study aimed to compare, following a 2-week familiarization period, the impact of two consecutive very low energy intake days on endothelial function compared to two consecutive ad libitum eating days, one week apart. The secondary aim was to explore the effects of these two dietary conditions on plasma glucose. Our hypothesis was that endothelial function and fasting plasma glucose levels would improve following the severe energy restriction days.

## 2. Materials and Methods

### 2.1. Subjects

**Figure 1:**
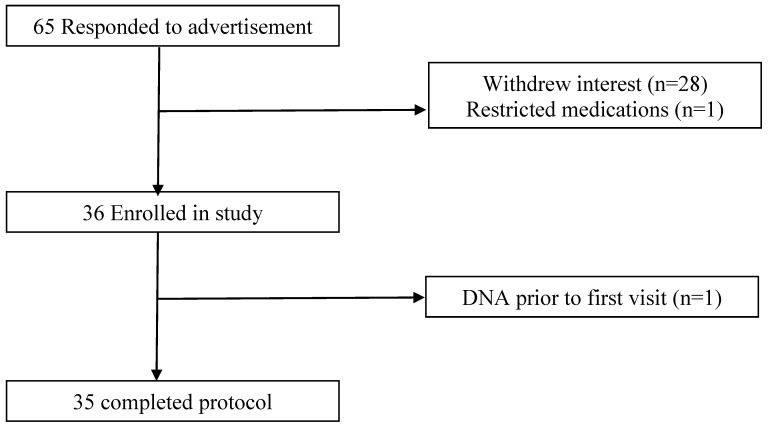
Consolidated standards of reporting trials diagram of the flow of subjects through the study.

Men and women aged between 18 and 70 years of age were recruited through flyer distribution on university notice boards, community advertisement through the Sansom Institute of Clinical Research website and personal contacts (Figure 1). Inclusion criteria were BMI (in kg/m^2^) ≥20, over the age of 18, weight stability in the preceding 6 months, not pregnant or breast feeding, and participants reporting that they were healthy. Individuals were not excluded if they were taking any other vitamin or mineral supplements, provided the dosage remained stable for the duration of the study. Exclusion criteria included having a metabolic disease, i.e., liver or kidney disease, treated hypertension, known and or treated hypercholesteremia, clinical CVD, and the inability to understand and or adhere to the study protocol. For female participants, data on the stage of menstrual cycle or if pre-menopausal was not collected.

### 2.2. Study Methods

**Figure 2:**
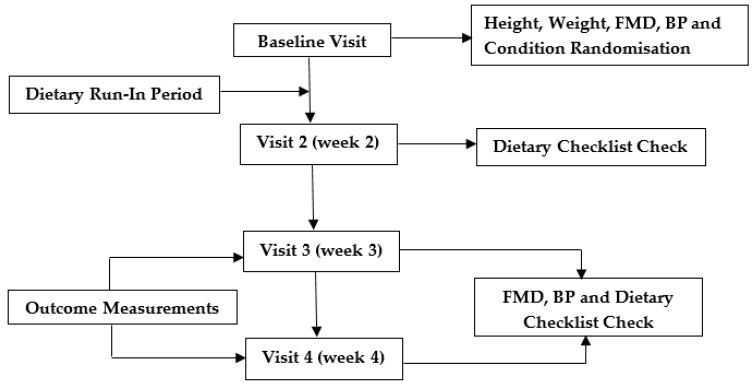
Schematic overview of study. Abbreviations: FMD: Flow mediated dilatation; BP: blood pressure.

In a randomized, single-blinded, crossover design, 35 participants completed the protocol over a 4-week period. Participants had a two-week dietary run-in period to ensure they were going to comply with the energy restriction protocol prior to visiting the clinic in weeks 3 and 4 following either 2 habitual eating days or 2 energy restricted days (Figure 2). After the baseline visit, each participant was assigned to their condition order (visiting following 2 energy restricted days either first or second) by using an online-generated balanced random-number allocation sequence (http://www.randomization.com). Participants were asked to fast (no food, ad libitum consumption water allowed) from 24:00 the night before and refrain from alcohol consumption 24 h before each visit to the clinic.

### 2.3. Dietary Intervention

Participants were asked to consume a very low energy diet (47% protein, 15% fat, 32% carbohydrate, 6% fibre, with ~300 mg sodium and ~1300 mg potassium) on two consecutive days per week along with 5 days of habitual eating. The diets were 500 calories for women and 600 calories for men. After receiving advice from a qualified dietitian, participants were asked to complete a weighed food checklist (scales supplied) on each very low-calorie day (four × two days—two days for each of the two-week run in period and two days each for the two testing weeks) and prepared all meals at home. Participants were asked to refrain from consuming alcohol or eating out on very low-calorie days and limit overall alcohol intake to no more two standard drinks across the 5 habitual eating days. They were also asked to keep physical activity consistent during the study. Participants acted as their own controls. At each study visit, participants met with the dietitian to discuss the completed weighed food checklists to determine on how many days during the trial they complied with the intervention. 

### 2.4. Weight and Height

Participants’ body height was measured (first visit only) whilst barefoot using a stadiometer (SECA 216 Stadiometer for Wall Mounting, SECA, Hamburg, Germany). Measurements were recorded to the nearest 0.1 cm. Body weight was recorded to the nearest 0.05 kg at all four visits using calibrated digital scales (SECA 703 Column, SECA, Hamburg, Germany). Participants were fasting, barefoot and in light clothing.

### 2.5. Flow Mediated Dilatation

Measurements of the right brachial artery in the longitudinal plane 5 cm above the antecubital fossa were performed by a single trained operator, using an 8.8 MHz linear array transducer (Samsung Medison MySono U6). Brachial artery diameter was measured via before-and-after forearm ischemia bought about by inflation of a sphygmomanometer cuff applied to the right forearm 2 cm below the olecranon process to 200 mmHg for 5 min. Readings were recorded pre compression (baseline), 30 s prior to cuff release, and then every 15 s after cuff release for 3 min [16]. Participants attended following an overnight fast and lay in a quiet, temperature-controlled room whilst images were being recorded. Images were saved for offline analysis. Measurements were taken at the baseline visit, and then following two very low-calorie days and two ad libitum eating days in a randomized order.

### 2.6. FMD Analysis

Ultrasound images were taken at a rate of 30 frames/s using screen-capture software (Debut Video Capture Software Professional V1.82; NCH Software, Greenwood Village CO, USA). All images were coded to blind the operator to the dietary condition at the time of analysis. Video frames were analyzed using edge detection software (Brachial Analyzer for Research V6.1.3; Medical Imaging Application LLC). A region of interest over a clear section of artery was selected, ensuring comparable images both pre-compression and post cuff release. The edge detection software was then used to execute frame-by-frame analysis and produce diameter measurements. The %FMD response was calculated as the percentage change from baseline to peak diameter of the artery. The coefficient of variation for this technique was 11% (*n* = 35).

### 2.7. Blood Pressure

Blood pressure was measured after participants had been seated at rest for at least 2 minutes. Measurements were taken using an automated sphygmomanometer (SureSigns VS3; Philips, North Ryde, Australia) while participants were fasting. Three consistent measurements (systolic within range of 10 mmHg and diastolic within range of 5 mmHg) taken 2 min apart were recorded and then averaged. 

### 2.8. Laboratory Analysis

Fasting blood samples were collected from a brachial vein into tubes with fluoride/EDTA for measurement of serum glucose. Samples of plasma were obtained by centrifuge at 4000 rpm for 10 min. Samples were stored at −80 °C until analyzed. Analysis was performed in a single set once all data collection was completed using a Konelab 20XT1 automatic analyzer (Thermo Electron Corporation, Louisville, CO, USA) with reagents from ThermoFisher Scientific (Melbourne, Australia).

### 2.9. Ethics

This study was approved by the University of South Australia’s Human Research Ethics Committee (approval number 0000036095). All participants gave written informed consent. This trial is registered at http://wwww.anzctr.org.au/ as ACTRN12617000510347.

### 2.10. Statistical Analysis

Based on a previous study [17], 32 people are required for a cross over study to have 80% power, *p* < 0.05 to detect at least a 2.0% FMD absolute change. Analyses was performed using SPSS Statistics^®^ 22 for Windows (SPSS Inc, Chicago, IL, USA). An ANOVA with repeated measures was run to analyze outcomes (with and without covariates including, age, diet order and BP). Associations between variables were evaluated with a Pearson’s correlation. Results are expressed as mean ± SDs unless otherwise stated.

## 3. Results

### 3.1. Subjects

Thirty-five participants competed the study. Baseline characteristics are outlined in Table 1.

### 3.2. Weight Loss

Dietary compliance was assessed via weighed food checklists recorded on each restricted day performed during the study. One hundred percent (*n* = 35) were compliant with the diet. There was weight loss across the four-week period of the trial (−1.4 kg, 95% CI 1–1.8 kg, *p* < 0.001). Dietary analysis is shown in Table 2. Analysis is separated into men and women, as they were asked to consume different energy intakes on the restricted days (500 calories of women and 600 calories for men).

### 3.3. Brachial Artery Endothelial Function and Blood Pressure

There was no difference between the two eating states in either absolute change in artery diameter or percentage change (Table 3). Baseline BMI and baseline FMD or FMD following the two eating states were not correlated. Change in weight and FMD were not correlated. Age, diet order or BP as a covariate had no effect. Blood pressure values did not change following the differing eating states. No significant correlation between blood pressure at each of the eating states and endothelial function was found.

### 3.4. Biochemical Analysis

There were no significant differences in glucose between the two dietary conditions (Table 3). There were no correlations between absolute FMD values and glucose.

## 4. Discussion

To our knowledge, this is the first study investigating the effects of severe energy restriction, as part of an intermittent energy restriction dietary pattern, on endothelial function as assessed by FMD.

A previous trial by Bhutani, et al. [18] investigated the effect of alternate day fasting (ADF) with and without endurance exercise, on endothelium-dependent flow mediated dilatation (FMD) over a 12-week time frame. There was no difference between groups at week 12, but it was unclear if measurements were taken following a fast or ad libitum eating period. 

Whilst not the primary outcome, it is important to recognise that the role of the weight loss experienced during the trial may have had on the overall FMD results. A meta-analysis conducted in 2015 showed weight loss significantly improved fasting FMD in adults (*n* = 265), with a reduction of 10 kg in body weight increasing fasting FMD by 1.11% [5]. However, this effect may depend on the type of weight loss treatment and dietary composition. Given the weight loss in this trial of 1.4 kg over 4 weeks, the effect of the weight difference between FMD measurements would be negligible. 

Reductions in LDL cholesterol (LDL-C) and fasting plasma glucose have been associated with improvements in FMD [19]. In the present study, we hypothesized that fasting glucose levels would be lower directly following the 2 days of energy restriction, resulting in an increased FMD. Previous studies in non-diabetics have highlighted that acute hyperglycaemia reduces endothelium-dependent vasodilation [20,21,22]. Several potential mechanisms suggested included reduced nitric oxide bioavailability [23,24], formation of advanced glycosylation end products [25] and chemical inactivation of nitric oxide by glucose [26]. Rodriguez, et al. [27] reported that fasting plasma glucose had an inverse, linear and independent correlation with endothelial function as measured by FMD with a 0.24% decrease in FMD for each 10mg/dl increase in fasting plasma glucose. Whilst FMD was not used as an outcome measurement, Antoni, Johnston, Collins and Robertson [11] assessed the effects of total and partial energy restriction on postprandial metabolism. Results showed that glucose iAUC response to a test meal (over a period of 6 h) was significantly lower following total (100%) energy restriction relative to the isoenergetic control diet (*p* = 0.015) and partial (75%) energy restriction. Compared to the isoenergetic control diet, fasting glucose levels were significantly lower following 1 day of partial (75%) or total (100%) energy restriction. The differing study design and collection time-points of this trial, as well as the population group studied (overweight or obese) may account for the conflicting results seen in the current study. No change to fasting plasma glucose levels was seen in this population with a normal fasting glucose. 

Dietary composition may play an important role on the effect of the potential impact of not just weight loss but also other biochemical markers on FMD. Studies focusing on this to date have shown mixed results. Raitakari, et al. [28] had individuals follow a low calorie, low carbohydrate (70 g/day) diet, and showed a decreased in fasting plasma glucose concentrations but not LDL-C or weight loss. Conversely, Clifton, et al. [29] showed no reductions in fasting plasma glucose or LDL-concentrations with weight loss, with no changes in FMD after participants followed a higher energy and carbohydrate CHO diet (~6000 kJ, 20% fat, >55% CHO, >70 g CHO). Bergholm, et al. [30] reported a weight loss of 7 kg after following a moderate fat (30% energy), high CHO (30% energy), with or without orlistat treatment. However, only the orlistat treated group saw improvements in endothelial function and reductions in LDL. Compared to Brook [31], where individuals lost a similar amount of weight on an energy restricted diet (30% energy as fat), reductions in LDL-C showed no change to FMD. The type of fat present in the diet has also shown to have an impact, with impairment of FMD being observed in weight stable subjects with a high saturated fat diet compared to a high monounsaturated, polyunsaturated fat, or low fat diet [32]. Similarly, varying sodium and potassium levels in dietary interventions has been shown to influence FMD. It has been shown that, after 4 weeks, FMD was increased in hypertensive and obese subjects when salt intake was decreased by 4–6 g per day (−100 mmol Na) [33,34]. Dickinson, et al. [35] then showed that a smaller reduction in dietary salt intake of 3 g/day improved endothelial function in normotensive, overweight and obese adults (*p* < 0.05). Increased dietary potassium intake from fruit and vegetables was shown to improve FMD within 1 week in healthy men and women (*p* = 0.03) [36], and a 36-mmol potassium meal has been shown to attenuate postprandial reduction in FMD [37]. Further to this, it has been shown that the addition of potassium to a high-sodium meal improved the sodium-induced post meal reduction in endothelial function as assessed by FMD [38]. Continued research into the impacts of varying dietary composition, as well as length of time following the protocol prior to testing, on FMD would be beneficial.

A review by Dharmashankar and Widlansky [39] found that high blood pressure levels lead to increased oxidative stress and inflammation, resulting in decreased endothelium dependent vasomotor dysfunction. However, in this study blood pressure was normal at baseline and did not change after either intervention.

We acknowledge that there are several limitations to this study. Firstly, the relatively healthy population sampled may have limited the ability to see changes in both biochemical and endothelial function changes. As the FMD measurements were taken following at least 8 h of fasting, the addition of 2 days of modified fasting may be a relatively weak additional stimulus. Lastly, we did not adjust for potential effects of the menstrual cycle, but gender had no influence on the result. In conclusion, this study showed that two consecutive days of energy restriction has no positive short-term effects on endothelial function.

## 5. Conclusions

In conclusion, this pilot study showed that two consecutive days of energy restriction has no effect on endothelial function, indicating that intermittent energy restriction in the form of the 5:2 diet strategy has no negative implications on the pathogenesis of CVD in a healthy population.

## Figures and Tables

**Table 1:** Baseline characteristics of participants ^1^.

Characteristic	Value
**Age, y**	46.5 ± 17.5
**Height, m**	1.7 ± 0.1
**Weight, kg**	72.1 ± 16.7
**BMI, kg/m^2^**	26.0 ± 5.5
**SBP, mm Hg**	118 ± 16
**DBP, mm Hg**	75 ± 11
**Baseline diameter, mm**	4.1 ± 1.0
**Brachial artery diameter change, mm**	0.3 ± 0.1
**FMD, %**	8.2 ± 2.4
**Glucose, mmol/L**	5.2 ± 0.8

^1^ All values are mean ± SD. *n* = 35 (25 women and 10 men). BMI, body mass index; DBP, diastolic blood pressure; FMD, flow mediated dilatation; SBP, systolic blood pressure.

**Table 2:** Dietary analysis of weight food checklists from restricted days (mean ± SD).

Nutrient	Male	Female
**Energy (cal)**	442 ± 104	369 ± 71
**Protein (g)**	34 ± 6	29 ± 6
**Total fat (g)**	12 ± 6	12 ± 5
**Saturated Fat (g)**	4 ± 3	4 ± 2
**Carbohydrate (g)**	43 ± 13	31 ± 9
**Sugars (g)**	28 ± 5	24 ± 7
**Fibre (g)**	14 ± 6	11 ± 4
**Sodium (mg)**	387 ± 222	392 ± 245
**Potassium (mg)**	1452 ± 261	1258 ± 220
**Iron (mg)**	4 ± 1	3 ± 1
**Zinc (mg)**	3 ± 1	3 ± 1
**Magnesium (mg)**	127 ± 37	99 ± 21
**Calcium (mg)**	355 ± 106	272 ± 74

**Table 3:** All outcome variables for each eating state for compliers to the protocol ^1^.

	2 ER Days	2 HB Days	*p*-Value ^2^
**Baseline diameter, mm**	4.0 ± 0.9	4.0 ± 0.8	0.7
**Absolute change in artery diameter, mm**	0.3 ± 0.02	0.3 ± 0.02	0.5
**FMD, %**	8.6 ± 0.4	8.3 ± 0.5	0.7
**SBP, mm Hg**	115 ± 12	118 ± 15	0.2
**DBP, mm Hg**	74 ± 9	73 ± 11	0.8
Glucose, mmol/L *	5.0 ± 0.7	5.1 ± 0.6	0.2

^1^ All values are mean ± SDs. *n* = 35 (25 women and 10 men). * Blood samples available *n* = 33. 2 HB days, 2 habitual eating days; 2 ER days, 2 energy restricted days; DBP, diastolic blood pressure; FMD, flow mediated dilatation; SBP systolic blood pressure; ^2^ Eating state effect.
